# Supplementary material for: STalign: Alignment of spatial transcriptomics data using diffeomorphic metric mapping
Source: Nat Commun. 2023 Dec 8;14:8123. doi: 10.1038/s41467-023-43915-7 (PMC10709594; doi:10.1038/s41467-023-43915-7)
Supplement: Supplementary file 1 — Supplementary Information [file 41467_2023_43915_MOESM1_ESM.pdf]

Supplementary Information for

STalign: Alignment of spatial transcriptomics data using diffeomorphic metric mapping

Kalen Clifton[1,2,*], Manjari Anant[1,3,*], Gohta Aihara[1,2], Lyla Atta[1,2], Osagie K. Aimiuwu[4], Justus M. Kebschull[2,5], Michael I. Miller[2,5], Daniel Tward[6,7], Jean Fan[1,2,5]

[1] Center for Computational Biology, Whiting School of Engineering, Johns Hopkins University, Baltimore, MD, USA

[2] Department of Biomedical Engineering, Johns Hopkins University, Baltimore, MD, USA

[3] Department of Neuroscience, Johns Hopkins University, Baltimore, MD, USA

[4] University of North Carolina at Chapel Hill, Chapel Hill, NC, USA

[5] Kavli Neuroscience Discovery Institute, The Johns Hopkins University, Baltimore, MD, USA

[6] Department of Computational Medicine, University of California Los Angeles, Los Angeles, CA, USA

[7] Department of Neurology, University of California Los Angeles, Los Angeles, CA, USA

* These authors contributed equally

Correspondence should be addressed to:

Jean Fan (jeanfan@jhu.edu) and Daniel Tward (dtward@mednet.ucla.edu)

Supplementary Tables

Slice 1	
RSP_L/ RSP_R	Turning point of retrosplenial area (left/right)
SCzo	Center of "dip" in superior colliculus
POST_L/ POST_R	Ventral/medial-most part of postsubiculum (left/right)
P	Follow the outline of the midbrain to the most ventral part; should be fairly center (point is actually a part of the pons)
DG_L/ DG_R	Ventral-most tip of dentate gyrus (left/right)
ENTm1_L/ENTm1_R	Tip of transition between entorhinal area, medial part, layer 1 and HATA (left/right)
CTXpl_L/CTXpl_R	Lateral-most part of cortical plate (left/right)
Slice 2	
RSPd1_L/RSPd1_R	Turning point for retrospenial area, dorsal part, layer 1 (left/right)
V3	Ventricle 3; in line with RSPv1 (the two most dorsal lines) and with the most ventral part of hippocampal formation (sunglasses)
CA3_L/CA3_R	Turing point of lateral-most part of CA3 (left/right)
ARH	Center of arcuate hypothalamic nucleus
VPL_L/VPL_R	Lateral-most part of ventral posterolateral nucleus of the thalamus (had to extrapolate in some areas to keep smooth curve) (left/right)
ec_L/ec_R	Follow the corpus callosum body (ccb) to the lateral-most part where it becomes the external capsule (should be slightly ventral to VPL points) (left/right)
dg_L/dg_R	Dentate gyrus; follow hippocampal formation from dorsal to ventral until distinct color change. Point should be at the last part of the initial color (centered at the beginning of what looks like an alligator's mouth) (left/right)
TH	Ventral-most part of thalamus
Slice 3	
MOs1_L/MOs1_R	Turning point for secondary motor area, layer 1 (left/right)
ccb	Center of corpus callosum body in line with ACAd/ACAv (two most dorsal lines)
CP_L_T	Top rightmost part of caudoputamen
CP_R_T	Top leftmost part of caudoputamen
och	Center of optic chiasm, in line with ccb point
CP_L_L/CP_R_L	Most lateral point of caudoputamen (left/right)
OT_L_L/OT_R_L	Most lateral point of olfactory tubercle (left/right)
OT_L_M/OT_R_M	Most medial point of olfactory tubercle (left/right)

Supplementary Table 1. Description of manually placed landmark locations on ST datasets of the mouse brain.

Jupyter notebook	STalign.LDDMM niter	GPU runtime	CPU runtime
merfish-visium-alignment-with-point-annotator.ipynb	200	GPU times: user 5min 6s, sys: 22.8 s, total: 5min 29s<br><br>Wall time: 5min 3s	CPU times: user 35min 57s, sys: 2min 43s, total: 38min 41s<br><br>Wall time: 9min 50s
merfish-merfish-alignment.ipynb	10000	GPU times: 32min 43s, sys: 10.9s, total: 32min 54s<br><br>Wall time: 22min 47s	CPU times: user 2h 39min 42s, sys: 31min 2s, total: 3h 10min 45s<br><br>Wall time: 1h 27min 7s
xenium-xenium-alignment.ipynb	200		CPU times: user 43.2 s, sys: 5.2 s, total: 48.4 s<br><br>Wall time: 41.1 s
xenium-heimage-alignment.ipynb	2000		CPU times: user 3min 22s, sys: 24.2 s, total: 3min 47s<br><br>Wall time: 3min 16s
xenium-starmap-alignment.ipynb	4000		CPU times: user 13min 36s, sys: 1min 52s, total: 15min 28s<br><br>Wall time: 14min
merfish-allen3Datlas-alignment.ipynb	2000		CPU times: user 3h 24min 29s, sys: 21min 43s, total: 3h 46min 13s<br><br>Wall time: 1h 27min 45s
starmap-allen3Datlas-alignment.ipynb	800		CPU times: user 2h 36min 26s, sys: 1h 16min 5s, total: 3h 52min 32s<br><br>Wall time: 13min 27s
heart-alignment.ipynb	1000		CPU times: user 33min 13s, sys: 1min 56s, total: 35min 10s<br><br>Wall time: 3min 53s

Supplementary Table 2. Runtime estimates for the STalign.LDDMM and the STalign.LDDMM_3D_to_slice functions for different ST data alignments.

Supplementary Figures

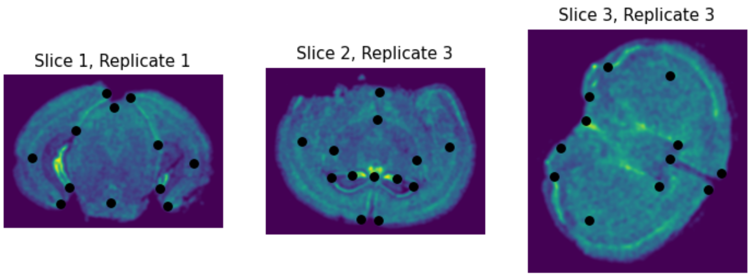

Supplementary Figure 1. Manually placed landmark locations on ST datasets for one representative biological replicate spanning 3 different locations with respect to bregma.

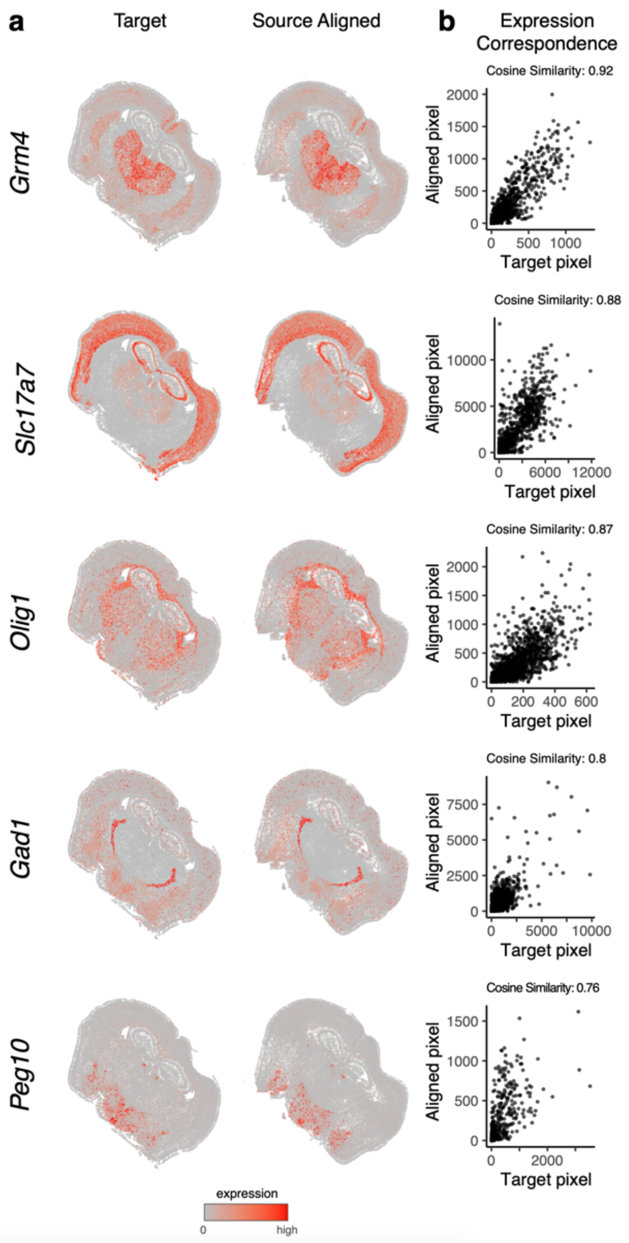

Supplementary Figure 2. ***Additional examples of MERFISH to MERFISH alignment for spatially patterned genes.*** **a.** Correspondence of gene expression spatial organization between the target and aligned source for select spatially patterned genes. **b.** Transcript counts in the target compared to the aligned source at matched pixels for select genes with cosine similarities between transcript counts in target versus aligned source marked.

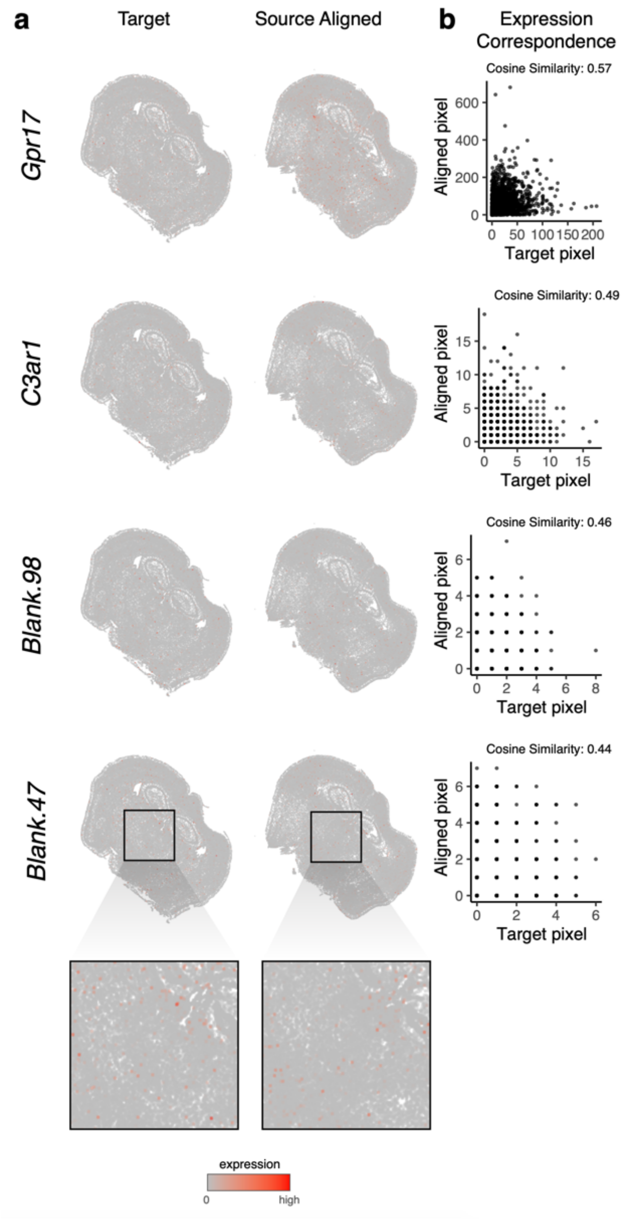

Supplementary Figure 3. ***Additional examples of MERFISH to MERFISH alignment for non-spatially patterned genes.*** **a.** Correspondence of gene expression spatial organization between the target and aligned source for select non-spatially patterned genes (inset displays cells at higher magnification). **b.** Transcript counts in the target compared to the aligned source at matched pixels for select genes with cosine similarities between transcript counts in target versus aligned source marked.

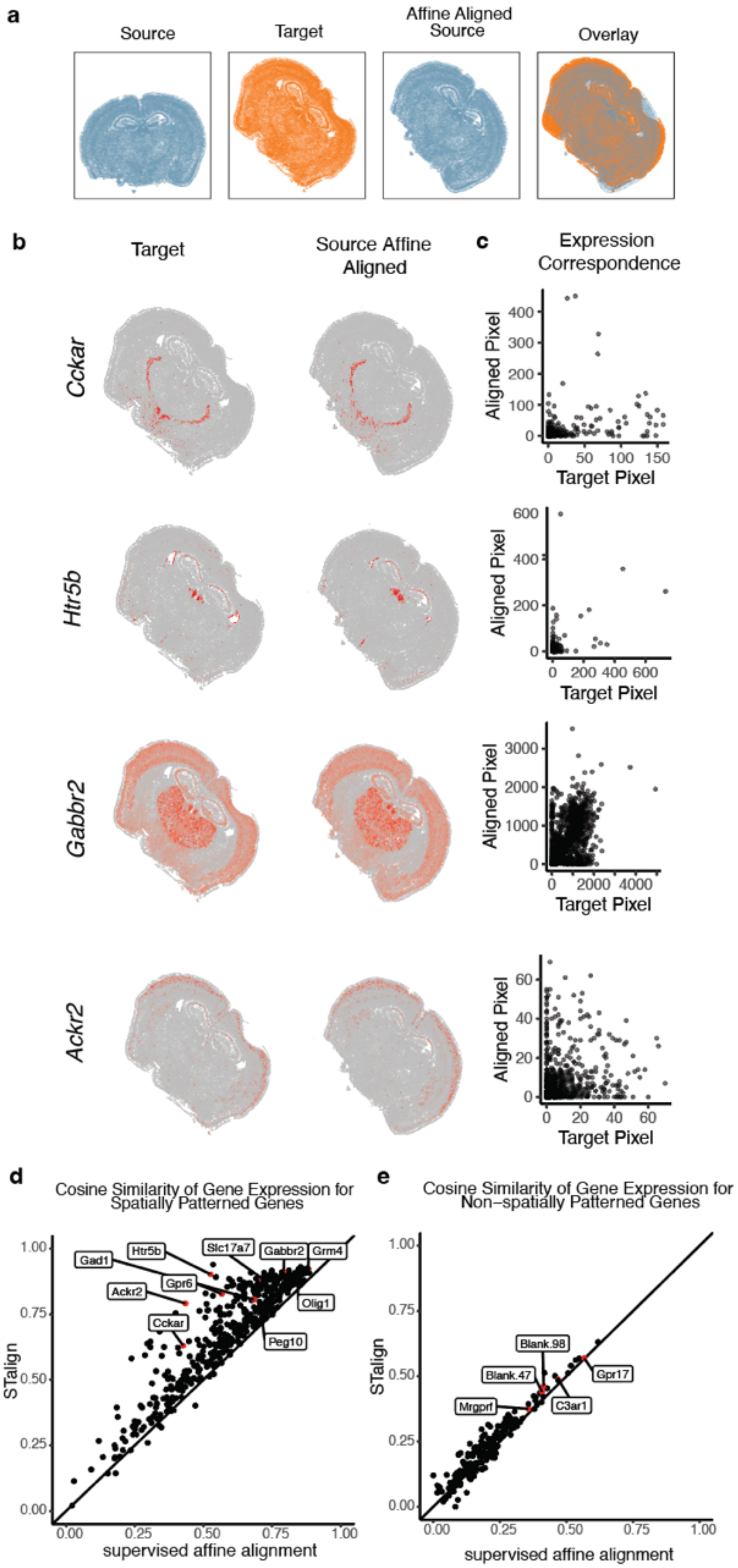

a
Source
Target
Affine Aligned Source
Overlay
b
Target
Source Affine Aligned
c
Expression Correspondence
Cckar
Htr5b
Gabbr2
Ackr2
Aligned Pixel
Target Pixel
d
Cosine Similarity of Gene Expression for Spatially Patterned Genes
STalign
supervised affine alignment
Gad1
Htr5b
Slc17a7
Gabbr2
Grm4
Gpr6
Ackr2
Olig1
Cckar
Peg10
e
Cosine Similarity of Gene Expression for Non−spatially Patterned Genes
Blank.98
Blank.47
Gpr17
Mrgprf
C3ar1

Supplementary Figure 4. ***Evaluation of STalign against supervised affine alignment.*** **a.** Spatial agreement of target and source that has been aligned based on a simple affine transformation based on manually placed landmarks. **b**. Correspondence of gene expression spatial organization between the target and supervised affine aligned source for select spatially patterned genes. **c.** Transcript counts in the target compared to the supervised affine aligned source at matched pixels for select genes: *Cckar*, *Htr5b*, *Gabbr2* and *Ackr2*. **d**. Cosine similarities between transcript counts in target versus aligned source for STalign compared to affine alignment for 457 spatially patterned genes. (mean difference = 0.09) Genes featured in Supplementary Figure 4b-c, Figure 2a-c, and Supplementary Figure 2 are highlighted. **e.** Cosine similarities between transcript counts in target versus aligned source for STalign compared to affine alignment for 192 non-spatially patterned genes. (mean difference = 0.02) Genes featured in Figure 2d-f and Supplementary Figure 3 are highlighted.

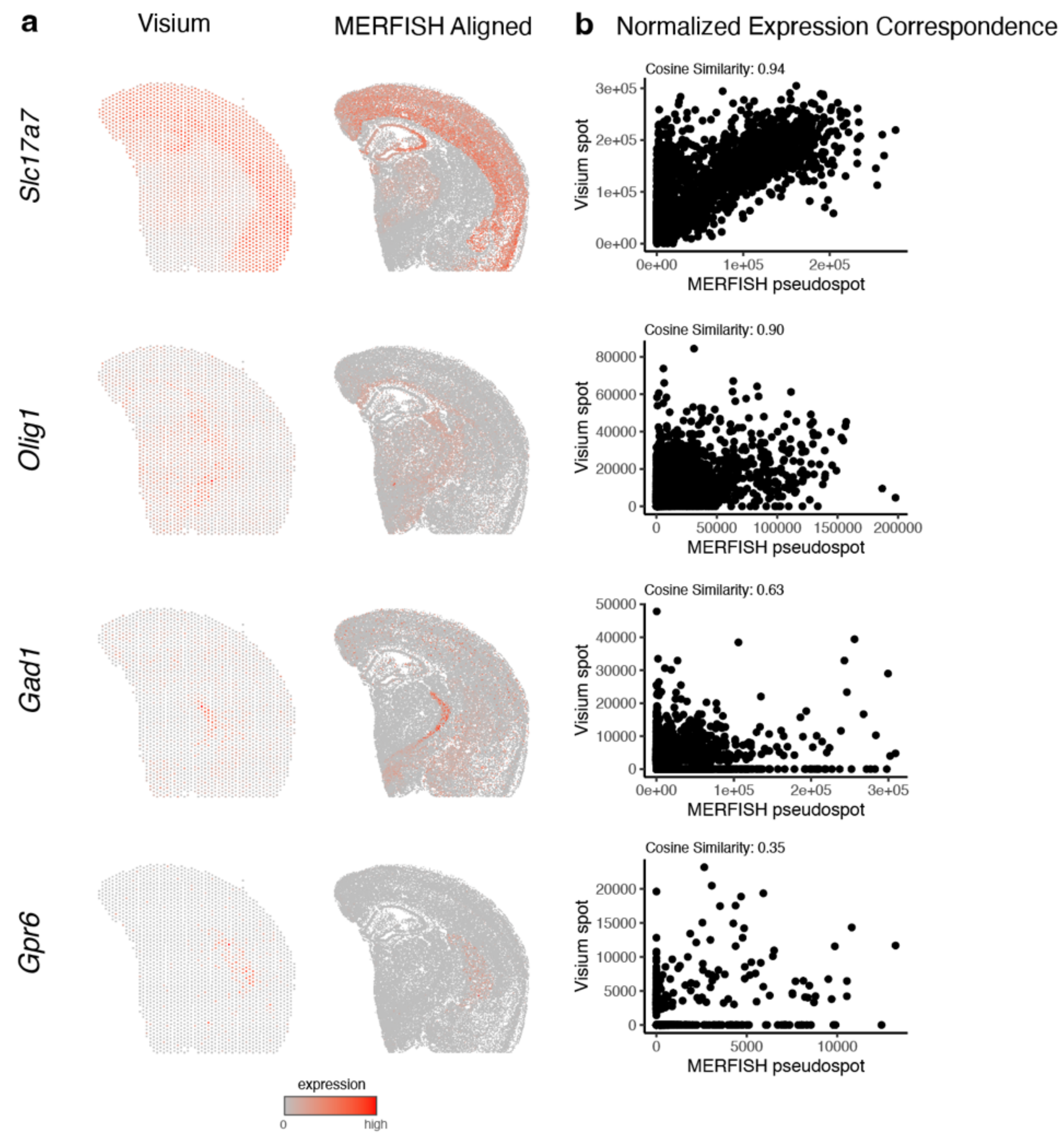

Supplementary Figure 5. ***Additional examples of MERFISH to Visium alignment for spatially patterned genes.*** **a.** Correspondence of gene expression spatial organization between the target and aligned source for select spatially patterned genes. **b.** Normalized gene expression in the Visium target compared to the aligned MERFISH source at matched spots and pseudospots for select genes with cosine similarities between transcript counts in target versus aligned source marked.

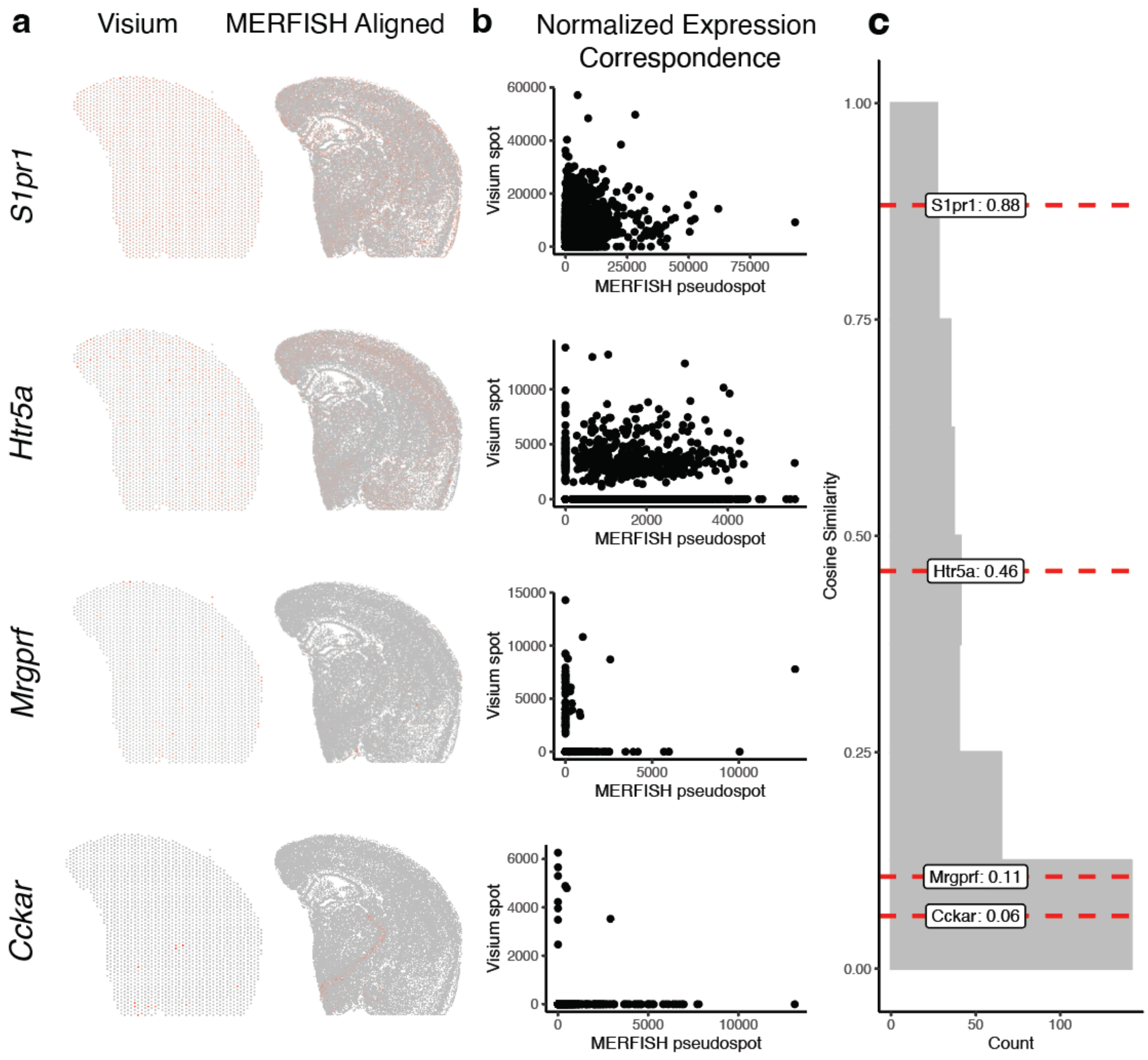

Supplementary Figure 6. ***Examples of MERFISH to Visium alignment for spatially non-patterned genes.*** **a.** Correspondence of gene expression spatial organization between the target and aligned source for select non-spatially patterned genes. **b.** Normalized gene expression in the Visium target compared to the aligned MERFISH source at matched spots and pseudospots for select non spatially patterned genes. **c**. Distribution of cosine similarities between normalized gene expression in the Visium target versus aligned MERFISH source at matched spots and pseudospots for 188 non-spatially patterned genes detected by both ST technologies with select genes marked.

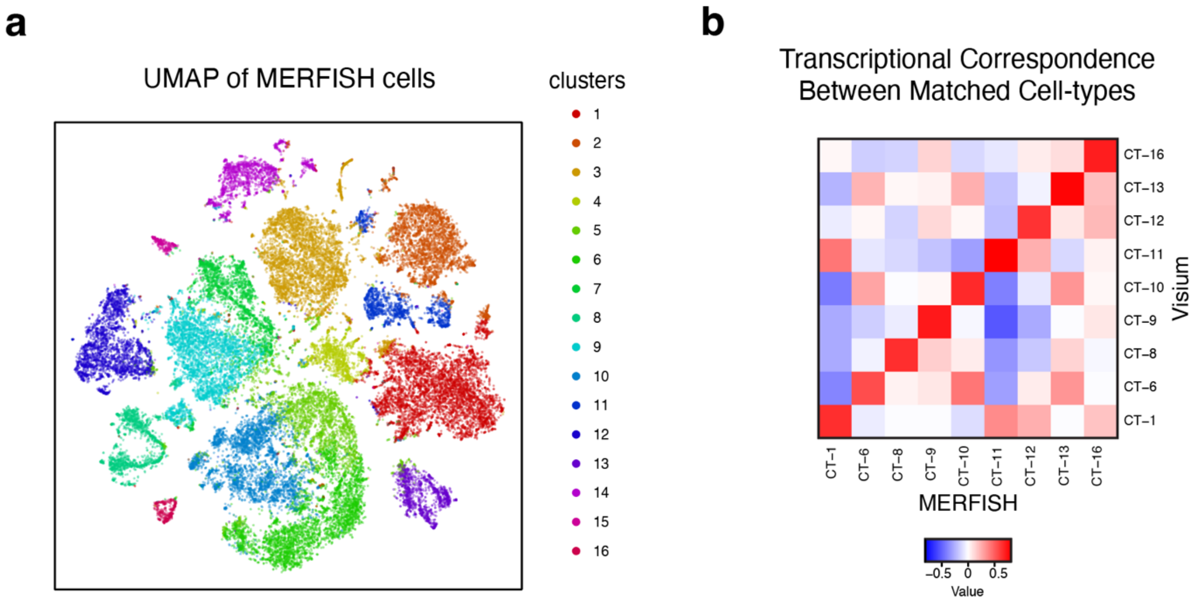

Supplementary Figure 7. ***Cell-type correspondence between clustering of MERFISH data and deconvolution of Visium data*****. a.** UMAP embedding of MERFISH cells colored by cluster. **b.** Heatmap of transcriptional correlation between the average expression for MERFISH clusters and deconvolved expression for Visium cell-types from STdeconvolve.

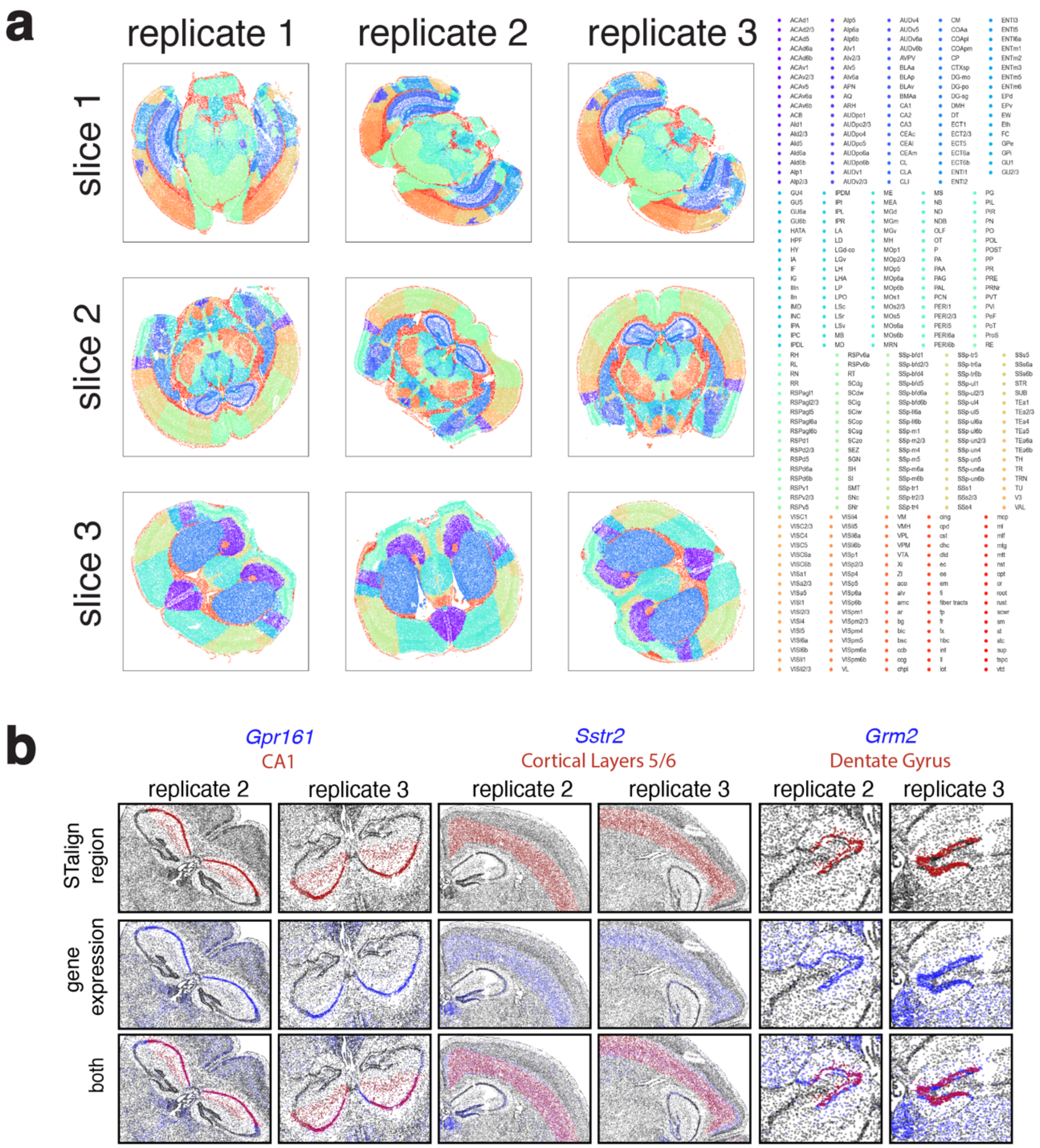

Supplementary Figure 8. ***STalign-annotated brain regions.*** **a.** Brain regions annotated by STalign, represented by different colors, for three biological replicates of three brain slices. **b.** Examples of genes (blue) expressed in brain regions (red) obtained through 3D alignment of MERFISH slices using STalign. Based on gene expression, brain region annotations show consistency and accuracy across replicates of brain slices.

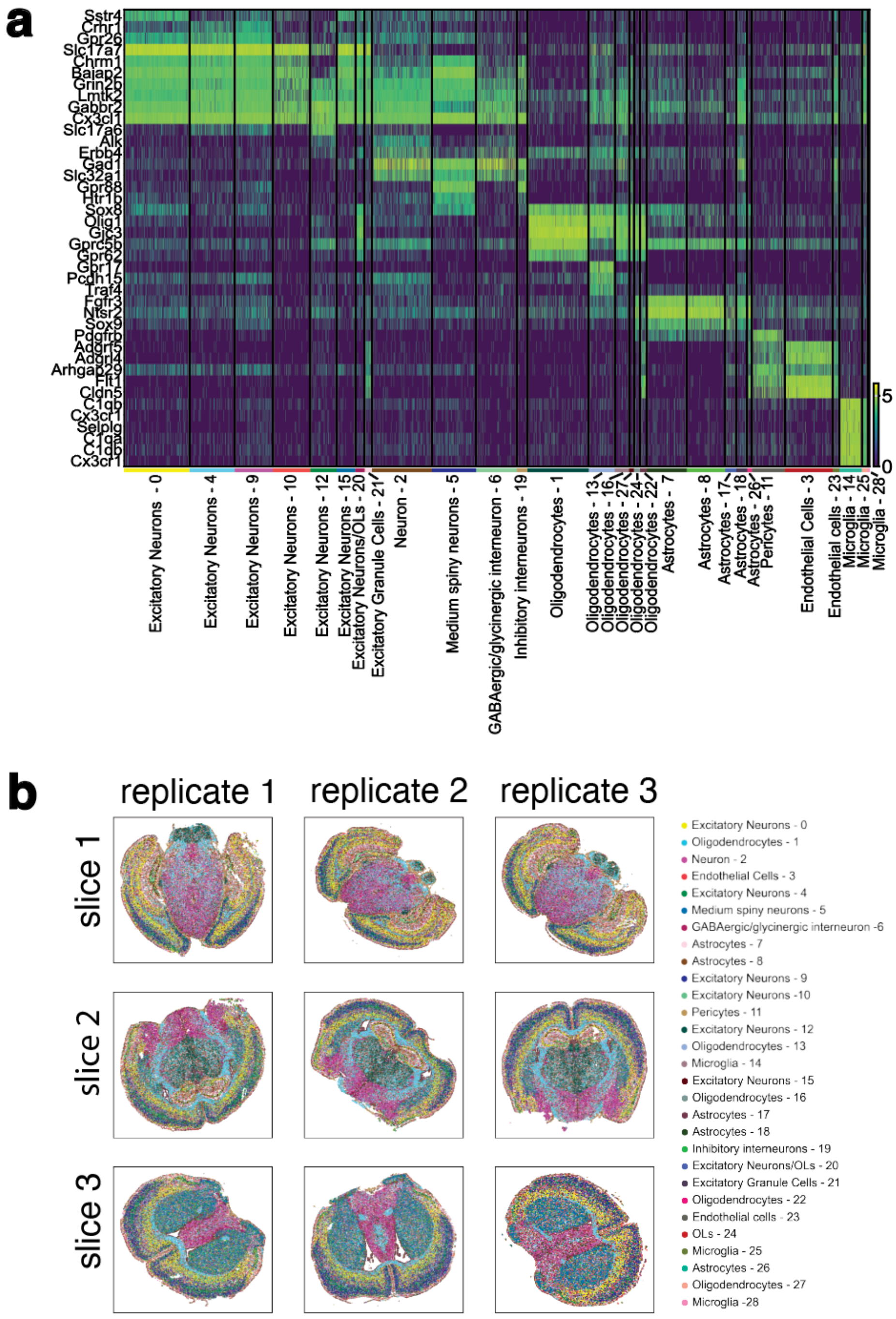

Supplementary Figure 9. ***Cell types in MERFISH dataset.*** **a.** Heat map of differentially expressed genes in cell types defined by Leiden clustering for all cells across 9 MERFISH datasets. **b.** Cell types defined by differential gene expression and Leiden clustering, represented by different colors, for three biological replicates of three brain slices.

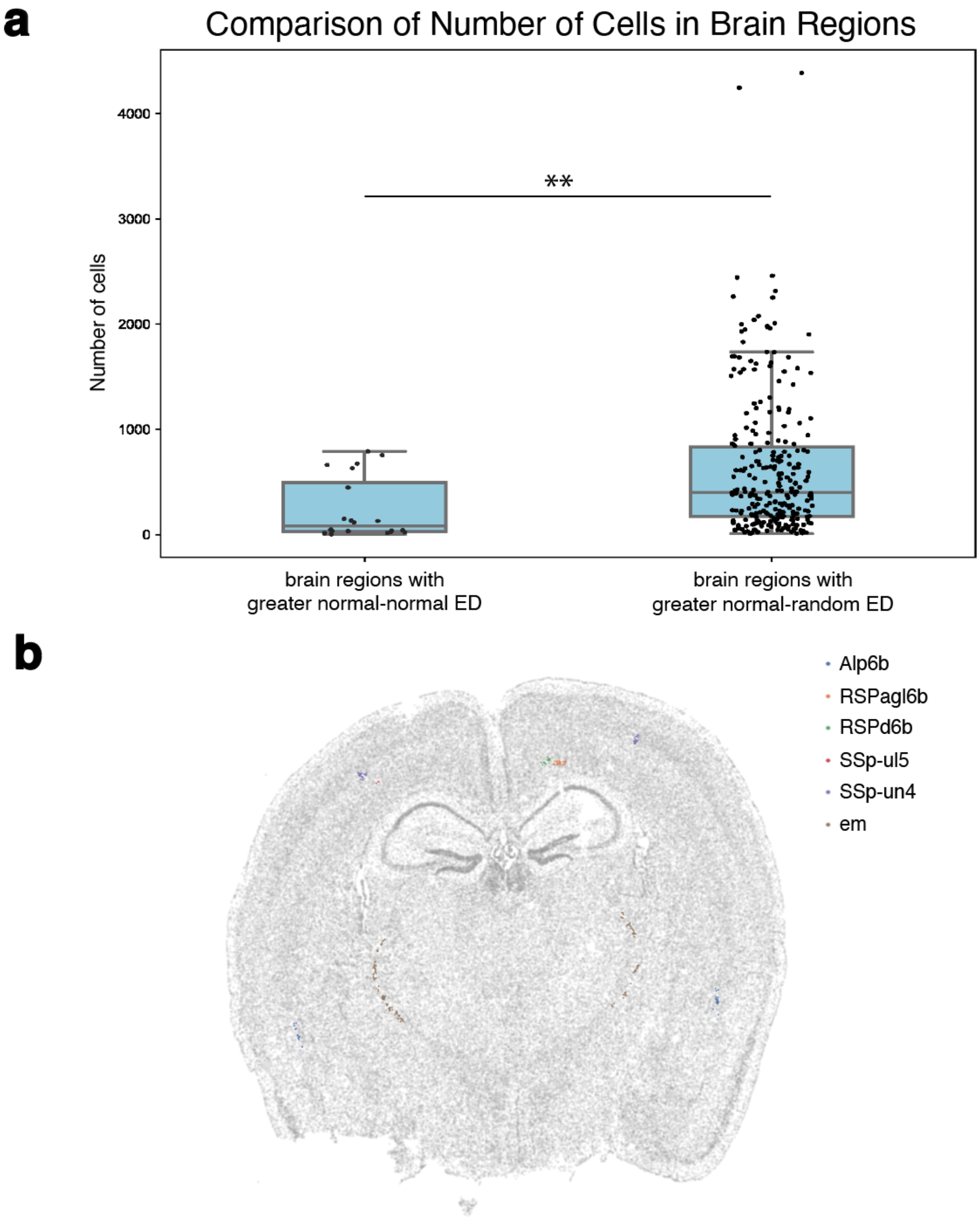

Supplementary Figure 10. ***Analyzing brain regions with lower cell-type compositional similarity between replicates compared to size-matched random regions.*** **a.** Distribution of number of cells in a brain region in Slice 2 Replicate 2 and Slice 3 Replicate 3 for which Euclidean-

distance (ED) was greater (left, n=10 brain regions) or smaller (right, n=131 brain regions) between replicates compared to matched randomly demarcated brain regions (center line, median; box limits, upper and lower quartiles; whiskers, 1.5x interquartile range; points, outliers; **, statistically significant p-value = 0.002) **b.** Representative MERFISH dataset (Slice 2 Replicate 3) of brain regions in the 'Greater normal-normal ED' suggestive of lower cell-type compositional similarity between replicates compared to size-matched random regions from (a) which were under 50μm in at least one dimension.

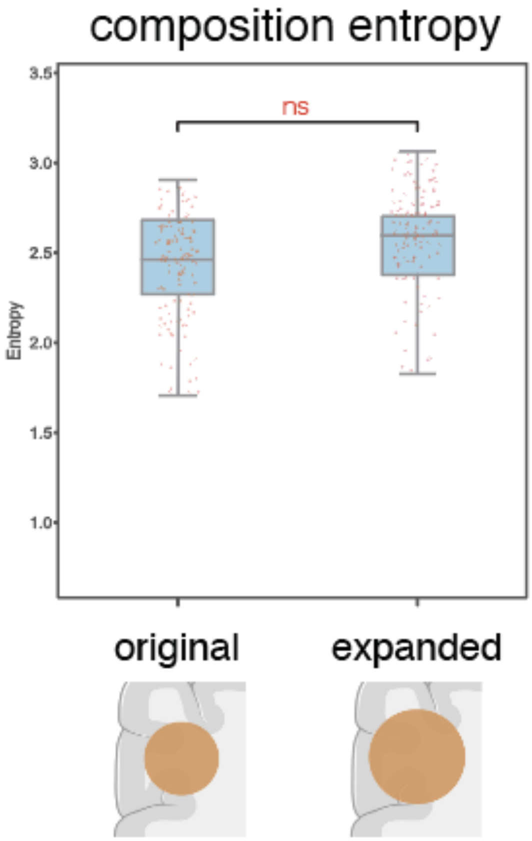

Supplementary Figure 11. ***Entropy for size-matched and expanded random brain regions.*** Non-significant (ns) difference between distribution of cell-type composition entropy for randomly demarcated brain regions that were matched in size for STalign-annotated brain regions (left) versus regions expanded by 100 nearest neighbors (~100µm) (center line, median; box limits, upper and lower quartiles; whiskers, 1.5x interquartile range; all data points shown, n=148 brain regions; p-value = 0.12). Created with Biorender.com.

Supplementary Notes for STalign: Alignment of spatial transcriptomics data using diffeomorphic metric mapping

Kalen Clifton[1,2,*] Manjari Anant[1,3,*], Gohta Aihara[1,2], Lyla Atta[1,2], Osagie K. Aimiuwu[4] Justus M. Kebschull[2,5] Michael I. Miller[2,5] Daniel Tward[6,7] Jean Fan[1,2,5]

[1]Center for Computational Biology, Whiting School of Engineering, Johns Hopkins University, Baltimore, MD, USA
[2]Department of Biomedical Engineering, Johns Hopkins University, Baltimore, MD, USA
[3]Department of Neuroscience, Johns Hopkins University, Baltimore, MD, USA
[4]University of North Carolina at Chapel Hill, Chapel Hill, NC, 27599
[5]Kavli Neuroscience Discovery Institute, The Johns Hopkins University, Baltimore, MD, USA
[6]Department of Computational Medicine, University of California Los Angeles, Los Angeles, CA, USA
[7]Department of Neurology, University of California Los Angeles, Los Angeles, CA, USA

1 Cell density data model

For single-cell resolution spatially resolved transcriptomics data, we model the point sets of detected cells in the framework of varifold measures [1]. While the theory extends to more complex spaces of features, here we focus on image varifolds [2] by utilizing the locations of cells only, termed the *marginal space measure* (after marginalizing out features other than spatial location) as defined in [3].

Briefly, these space measures are weighted sums of Dirac δ distributions $\rho \doteq \sum_i^{N_q} w_i^\rho \delta_{x_i^\rho}$, where $x_i^\rho \in \mathbb{R}^D$ stores the spatial coordinate of the ith out of N_q cells, and $w_i^\rho \in \mathbb{R}$ stores its weight. In this work, cell positional data is two dimensional, so $D = 2$, and with some abuse of notation we sometimes write (x, y) instead of x.

We aim to evaluate the similarity between two single-cell resolution spatially resolved transcriptomics datasets, which we call a source and a target. Note other commonly used terms for source are: template, atlas, or moving image, while another commonly used terms for target is: fixed image. To compute distances between datasets, we embed their corresponding space measures ρ_S and ρ_T respectively in the dual of a Reproducing Kernel Hilbert Space V^* and use the standard operator norm (see for example [4]). For some choice of kernel function k, the norm squared is

$$\begin{aligned}\|\rho_S - \rho_T\|_{V^*}^2 = &\sum_{i_S, j_S}^{N_{\rho_S}} w_{i_S}^{\rho_S} w_{j_S}^{\rho_S} k(x_{i_S}^{\rho_S}, x_{j_S}^{\rho_S}) \\ &- 2 \sum_{i_S}^{N_{\rho_S}} \sum_{j_T}^{N_{\rho_T}} w_{i_S}^{\rho_S} w_{j_T}^{\rho_T} k(x_{i_S}^{\rho_S}, x_{j_T}^{\rho_T}) \\ &+ \sum_{i_T, j_T}^{N_{\rho_T}} w_{i_T}^{\rho_T} w_{j_T}^{\rho_T} k(x_{i_T}^{\rho_T}, x_{j_T}^{\rho_T}) \ . \end{aligned} \tag{1}$$

Here we chose k as a Gaussian with $k(x_i, x_j) = \exp\left(-\frac{1}{2}|x_i - x_j|^2/2\sigma^2\right)$, where $|\cdot|$ denotes the standard Euclidean norm, and σ is a user specified kernel width parameter.

The variables $w_{i_S}^{\rho_S}$, $w_{j_S}^{\rho_S}$, and $x_{i_S}^{\rho_S}, x_{j_S}^{\rho_S}$ corresponds to the weights and spatial coordinates of the ith and jth cells in the source, while $w_{i_T}^{\rho_T}$, $w_{j_T}^{\rho_T}$, $x_{i_T}^{\rho_T}, x_{j_T}^{\rho_T}$ correspond to the weights and spatial coordinates of the cells in the target. For simplicity, in the main paper we write (x^{ρ_S}, y^{ρ_S}) and (x^{ρ_T}, y^{ρ_T}) for source and target points respectively.

In STalign, we initialize weights to 1, though applying nonlinear deformations will modify these weights as discussed below in section 4.

2 Rasterization

Since computation of this norm is of quadratic complexity in the number of points, we turn to a more efficient representation for computing optimal transformations through rasterization. We can reduce the complexity of our calculations significantly by approximating our space measures through sampling a density signal on a regular grid (known as rasterization), rather than keeping a list of points and weights.

By defining $k^{\frac{1}{2}}$ such that $k^{\frac{1}{2}} * k^{\frac{1}{2}} = k$ (where $*$ refers to convolution), the above expression for norm squared (1) can be written as

$$\begin{aligned} \|\rho_S - \rho_T\|_{V^*}^2 &= \int \left| \sum_{i_S=1}^{N_{\rho_S}} w_{i_S}^{\rho_S} k^{\frac{1}{2}}(x - x_{i_S}^{\rho_S}) - \sum_{i_T=1}^{N_{\rho_T}} w_{i_T}^{\rho_T} k^{\frac{1}{2}}(x - x_{i_T}^{\rho_T}) \right|^2 dx \\ &= \|I^S - I^T\|_2^2 . \end{aligned} \tag{2}$$

Note that when k is a radially symmetric Gaussian, $k^{\frac{1}{2}}$ is also a radially symmetric Gaussian but with half the variance. Here we have defined the smooth density function

$$I(x) \doteq [k^{\frac{1}{2}} * \rho](x) = \sum_{i=1}^{N_\rho} w_i^\rho k(x - x_i^\rho) , \tag{3}$$

and $\|\cdot\|_2$ is the L_2 norm on functions.

Due to the smoothness introduced by $k^{\frac{1}{2}}$, these functions can be accurately discretized by sampling them on a uniform pixel grid at a resolution rate defined by the user and comparable in size to σ.

Without rasterization, evaluating the function I at a given point would involve a sum over every x_i, an order N complexity operation. After rasterization the function I can be evaluated at any point in order 1 complexity using bilinear interpolation. This allows the norm to be evaluated by summing over a pixel grid in order P complexity (where P is the number of pixels), rather than a double sum over the points x_i in order N^2 complexity.

For example, MERFISH Slice 2 Replicate 3 has 85958 cells, and the rasterized dataset has $336 \times 256 = 86,016$ pixels. The Naive approach would involve 7,388,777,764 terms in the sum (pairs of cells), whereas in the rasterization approach there are only 86,016 terms in the sum (pixels). This is an approximately 86,000 times increase in efficiency which occurs for each iteration of optimization, ignoring the negligible time cost of rasterization itself, which occurs only once at the start of registration.

In this section we showed how a rasterized image I can be produced from a list of cell location, in a manner compatible with the theory of varifolds. However, our registration algorithm can be performed with any standard rasterized image type. For example, in the main manuscript we show examples where I^T is a red-green-blue image corresponding to a brightfield microscopy image of H&E stained tissue. How such images of different contrast profiles are handled is described in section 5.

3 Diffeomorphic transformation model

We estimate alignments between two rasterized datasets by applying a transformation $\phi : \mathbb{R}^D \to \mathbb{R}^D$. $\phi(x) \doteq A\varphi_1(x)$, the composition of two transformations: a diffeomorphism (a smooth differentiable transformation

with a smooth differentiable inverse) $\varphi_1 : \mathbb{R}^D \to \mathbb{R}^D$ generated in the Large Deformation Diffeomorphic Metric Mapping (LDDMM) framework [5], and an affine transformation A (i.e. a 3x3 matrix in homogeneous coordinates whose upper left 2x2 block is a linear transform and upper right 2x1 block is a translation vector). In this notation $A\varphi_1(x)$ denotes matrix multiplication of the matrix A and the vector φ_1 in homogeneous coordinates.

In the LDDMM framework a diffeomormphism is generated by integrating a time varying velocity field v_t over time $t \in [0,1]$, by solving the ordinary differential equation

$$\frac{d}{dt}\varphi_t = v_t(\varphi_t) \tag{4}$$

with initial condition $\varphi_0 = \text{id}$. For identifying alignments, we optimize over v_t rather than φ_1 directly, and to emphasize this dependence we use the superscript φ^v in the main text. Similarly, we use $\phi^{A,v}$ to emphasize the dependence of ϕ on both A and v_t. As long as v_t a smooth function of space, φ_1^v is guaranteed to be diffeomorphic. We enforce this through regularization as described below in section 5.

While this section described how we parameterize our transformations, next we need to describe how they act to deform our datasets, in order to use them in an optimization problem.

4 Action of transformations on datasets

The action of a transformation ϕ on a space measure dataset ρ moves the spatial coordinate of each cell, and adjusts the weight of each cell based on the transformation's Jacobian determinant.

$$\phi \bullet \rho = \phi \bullet \left(\sum_{i=1}^{N_\rho} w_i^\rho \delta_{x_i^\rho} \right) \tag{5}$$

$$= \sum_{i=1}^{N_\rho} w_i^\rho |d\phi(x_i^\rho)| \delta_{\phi(x_i^\rho)} \tag{6}$$

where $d\phi(x)$ denotes the matrix of partial derivatives of the map ϕ at the point x, and $|\cdot|$ represents the determinant of a matrix.

We note that the standard image action $[\phi \cdot I](x) = I(\phi^{-1}(x))$ has been well studied theoretically (as a left group action), computationally (in terms of its efficient implementation through interpolation), and application-wise (in terms of its use in a variety of image registration platforms e.g. [5]). This image action for continuous image functions is not appropriate for space datasets and therefore the image action does not match the measure action defined in (5). However, in the dense tissue limit, the continuous image action is consistent with the measure action of (5) as proven in [3]. Since the applications shown here provide a dense approximation, this aforementioned consistency motivates us to leverage the continuous image action for its computational advantages. We write the image action as follows:

$$[\phi \cdot I](x) = [\phi \cdot (k^{\frac{1}{2}} * \rho)](x) \tag{7}$$

$$= I(\phi^{-1}(x)) \tag{8}$$

$$= \sum_{i=1}^{N_\rho} w_i^\rho k^{\frac{1}{2}}(\phi^{-1}(x) - x_i^\rho) \tag{9}$$

$$\simeq \sum_{i=1}^{N_\rho} w_i^\rho |d\phi(x_i^\rho)| k^{\frac{1}{2}}(x - \phi(x_i^\rho)) \tag{10}$$

$$= [k^{\frac{1}{2}} * (\phi \bullet \rho)](x) \tag{11}$$

The approximate equality is accurate in our examples when $k^{\frac{1}{2}}$ is narrow relative to the smoothness of v_t and wide relative to the spacing between cells. The approximate equality would be exact if $k^{\frac{1}{2}}$ were a Dirac

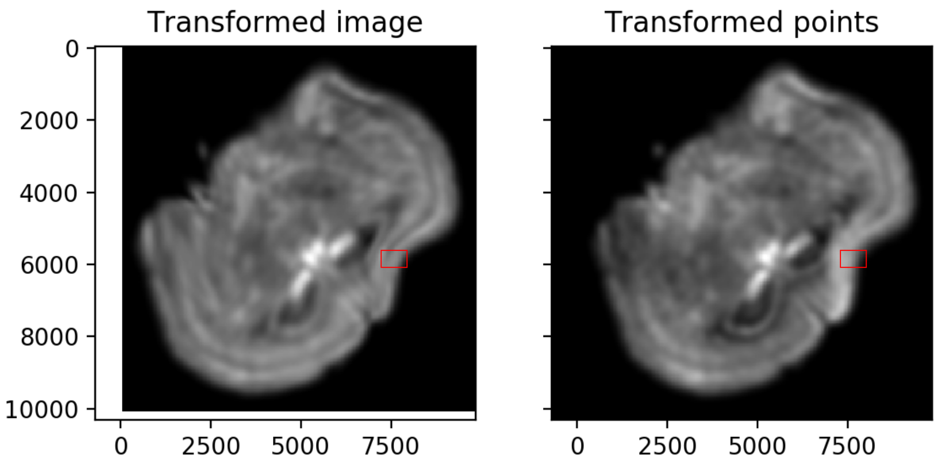

Supplementary Notes Figure 1: Rasterization followed by deformation with the image action (left), versus naively deforming the positions of cells followed by rasterization (right). Note the intensity changes that occur in regions of high deformation.

delta function. If cells are too far apart, a larger value of σ could be chosen. For a particularly sparse set of cells, a different method that does not include rasterization would be more appropriate, for example measure matching [6].

With this formulation, deformations can be applied to smooth density images I using interpolation in order P (number of pixels) complexity. Supplementary Notes Figure 1 illustrates the importance of the Jacobian factor. Without including this factor, transforming a density image alters its brightness, which is typically undesirable: a bigger organ tends to have more cells with the same cell density, rather than the same number of cells with a lower density.

5 Image registration

We compute a spatial alignment between two ST datasets by minimizing the sum of two objective functions: a regularization term R, and a matching term M_θ,

$$E(A, v) = R(v) + M_\theta(\phi^{A,v} \cdot I^S, I^T) \tag{12}$$

which we define below. Note that the computation of I is described in section 2, the parameterization of $\phi^{A,v}$ is described in section 3, and the action $\phi^{A,v} \cdot I^S$ is described in the section 4. Recall that ϕ depends on both the velocity field v and the affine transform A.

Following the LDDMM framework, we regularize our diffeomorphism via

$$R(v) = \frac{1}{2\sigma_R^2} \int_0^1 \int_{\mathbb{R}^D} |(\mathrm{id} - a^2\Delta)^p v_t(x)|^2 dx dt \tag{13}$$

where id is an identity matrix, σ_R^2 is a user tunable parameter that adjusts balance between matching accuracy and regularization, where large values correspond to less regularization and higher matching accuracy, and small values correspond to more regularization and lower matching accuracy. Δ is the Laplacian, a is a constant with units of length that controls spatial smoothness, and $p = 2$ is a power that must be large enough to guarantee that results are diffeomorphisms [7]. Note that small values of a may be overfitting of noise whereas large values of a may lead to low accuracy. In practice, we chose a value of a based on the spatial smoothness of deformations that we believe to be realistic. We then consider several values of σ_R^2 (starting with a value provided by one of our online examples), and chose the one that achieves a reasonable balance between regularization and accuracy.

Our matching takes the form of [8]

$$M_\theta(\phi^{A,v} \cdot I^S, I^T) = \frac{1}{2\sigma_M^2} \int_{\mathbb{R}^D} |f_\theta([\phi^{A,v} \cdot I^S](x)) - I^T(x)|^2 W_M(x) dx \ . \tag{14}$$

where σ_M^2 is a user tunable parameter that describes the amount of noise in our imaging data (see description of Gaussian Mixture modeling below).

Note that I^T need not correspond to a smooth density image as defined in section 2. For example, we include the case where it is a red green blue image corresponding to an H&E stain.

The function f_θ is a transformation of image contrast with unknown parameters θ. We use a polynomial for f_θ, in which case the minimizing parameters θ can be found exactly by solving a weighted least squares problem. The purpose of this transformation is to model differences in contrast between images from the same modality due to calibration issues; and contrast/color differences between different modalities.

In this work we found that first order polynomials were sufficient for accurate image registration. In other work in neuroimaging we have used 3rd order polynomials, which have enough degrees of freedom to map the intensity of gray matter, white matter, and background to arbitrary intensities [8]. Because there are many more pixels than degrees of freedom, it is unlikely that these polynomials will overfit the observed data I^T. However, depending on the initialization of transformation parameters this is possible: if tissue in I and I^T do not overlap at all, parameters θ may be estimated to zero out imaging information and transform I into a constant function that looks like background only.

The term W is a positive weight that represents the probability that a given pixel in the target image can be matched accurately to one in the source image. For example, if tissue is missing in the target image but not the source image, pixels in the region of missing tissue would get a small weight. Similarly, if the target image included a signal not present in the source (e.g. a bright fluorescence signal).

To optimize E, we alternate between updating W_M with Gaussian mixture modeling, and jointly updating $(\theta, \phi^{A,v})$ with gradient based methods, using expectation maximization algorithm as discussed in [8]. Briefly, we use 3 classes in our Gaussian mixture model (pixels to be matched, background, and artifact). Each is modeled as a Gaussian random variable with an unknown mean (optionally the mean can be assumed known and specified as an input parameter), a known variance (specified as an input parameter), and an unknown prior probability. While the means for background and artifact are constant values, the mean for pixels to be matched is equal to $f_\theta([\phi^{A,v} \cdot I^S](x))$ and is a function of space. Parameters are estimated by standard Gaussian mixture modeling techniques, and $W_M(x)$ is computed as the posterior probability that the pixel at x belongs to the "pixels to be matched" class. If $g(x, \mu, \sigma^2)$ is a multivariate normal with mean μ and covariance σ^2 times identity, and π_i are prior probabilities for each class ($i \in$(matching, background, and artifact)), then

$$W_M(x) = \frac{\pi_M g(I^T(x), f_\theta([\phi^{A,v} \cdot I^S](x)), \sigma_M^2)}{\pi_M g(I^T(x), f_\theta([\phi^{A,v} \cdot I^S](x)), \sigma_M^2) + \pi_B g(I^T(x), \mu_B, \sigma_B^2) + \pi_A g(I^T(x), \mu_A, \sigma_A^2)} \tag{15}$$

In the above expression, note that the denominator shows a mixture of three Gaussians, and the numerator shows the first class in the mixture. In our code we also define W_B and W_A, which are posterior probabilities that a pixel belongs to the background or artifact classes. They are defined with same denominator as W_M, but with numerators corresponding to the mixture component for their class. Recall that updating ϕ corresponds to updating the affine transformation matrix A, and the velocity field v_t which generates the deformation φ_1 from (4).

After solving for the optimal transformation parameters A and v_t, a transformation and its inverse are constructed by solving (4) sampled on a regular grid, using Semi-Lagrangian techniques [9]. With $\phi(x) = A\varphi_1^v(x)$ and $\phi^{-1,A,v}(x) = \varphi_1^{-1,v}(A^{-1}x)$ computed, cell locations $x_{i_S}^{\rho_S}$ in the source image can be mapped into the target by calculating $\phi(x_{i_S}^{\rho_S})$ through linear interpolation. Similarly, a point $x_{i_T}^{\rho_T}$ in the target image can be mapped to the atlas by calculating $\phi^{-1}(x_{i_T}^{\rho_T})$ through linear interpolation.

6 Landmark Optimization Option

For improved robustness, our software allows users to input pairs of corresponding points in the source and target images. These points can be used either to initialize the affine transformation A through least squares

(steering our gradient based solution toward an appropriate local minima in this challenging nonconvex optimization problem); or can be used to drive the optimization problem itself by modifying to our objective function to be $E(A,v) = R(v) + M_\theta(\phi^{A,v} \cdot I^S, I^T) + P(\phi^{A,v}(X^S), X^T)$ such that

$$P(\phi^{A,v}(X^S), X^T) = \frac{1}{2\sigma_P^2} \sum_{i=1}^{N} |\phi^{A,v}(X_i^S) - X_i^T|^2 \tag{16}$$

where X_i^S and X_i^T are the ith point of N corresponding points in the source and target respectively and σ_P^2 is a user tunable parameter that adjusts balance between matching corresponding landmark points, matching images, and regularization, where large values correspond to less accuracy matching points and small values correspond to more accuracy matching points. Landmark based optimization in the LDDMM framework has been studied extensively (see for example [10]).

7 3D to 2D alignment

In addition to aligning spatially resolved transcriptomics datasets in which the cell positional information is 2D, we registered the 3D reconstructed Allen common coordinate framework (CCF) atlas (source) to each of the 9 MERFISH datasets (target). The image transformation is similar to the alignment discussed in the section 5 with a few exceptions:

It is important to note that all transformations are performed on the source 3D atlas. Since the 50 μm Nissl-stained Allen Brain Atlas CCF v3 was used as the source image, rasterization is not applied to the atlas. The affine transformation A for the 3D-2D alignment is a 4×4 3D matrix in homogeneous coordinates. The space of dense 3D images in the orbit of the atlas, are defined via diffeomorphisms

$$\phi : (x_1, x_2, x_3) \in \mathbb{R}^3 \to \phi(x) = (\phi_1(x), \phi_2(x), \phi_3(x)) \in \mathbb{R}^3 \tag{17}$$

The diffeomorphism $\phi \in D$ acts on the atlas to generate the orbit of imagery $\mathcal{I}$,

$$I \in \mathcal{I}, I = \phi \cdot I_{atlas} \ . \tag{18}$$

The velocity field v_t is still defined by (4), but $v_t \in \mathbb{R}^3$.

The image I^S in the matching term M represents the transformed source atlas evaluated at $z = 0$, to enable comparison in the same dimension between the source and the target images.

Supplementary References

[1] W. K. Allard, "On the first variation of a varifold," *Annals of mathematics*, vol. 95, no. 3, pp. 417–491, 1972.

[2] M. Miller, A. Trouvé, and L. Younes, "Image varifolds on meshes for map- ping spatial transcriptomics," *arXiv*, 2022.

[3] M. Miller, D. Tward, and A. Trouvé, "Molecular computational anatomy: Unifying the particle to tissue continuum via measure representations of the brain," *BME Frontiers*, vol. 2022, p. 9868673, 2022.

[4] J. Glaunès, A. Qiu, M. I. Miller, and L. Younes, "Large deformation diffeomorphic metric curve mapping," *International journal of computer vision*, vol. 80, no. 3, pp. 317–336, 2008.

[5] M. F. Beg, M. I. Miller, A. Trouvé, and L. Younes, "Computing large deformation metric mappings via geodesic flows of diffeomorphisms," *International journal of computer vision*, vol. 61, no. 2, pp. 139–157, 2005.

[6] J. Glaunes, A. Trouvé, and L. Younes, "Diffeomorphic matching of distributions: A new approach for unlabelled point-sets and sub-manifolds matching," in *Proceedings of the 2004 IEEE Computer Society Conference on Computer Vision and Pattern Recognition, 2004. CVPR 2004.*, vol. 2, pp. II–II, IEEE, 2004.

[7] P. Dupuis, U. Grenander, and M. I. Miller, "Variational problems on flows of diffeomorphisms for image matching," *Quarterly of applied mathematics*, pp. 587–600, 1998.

[8] D. Tward, T. Brown, Y. Kageyama, J. Patel, Z. Hou, S. Mori, M. Albert, J. Troncoso, and M. Miller, "Diffeomorphic registration with intensity transformation and missing data: Application to 3d digital pathology of alzheimer's disease," *Frontiers in neuroscience*, vol. 14, p. 52, 2020.

[9] A. Staniforth and J. Côté, "Semi-lagrangian integration schemes for atmospheric models—a review," *Monthly weather review*, vol. 119, no. 9, pp. 2206–2223, 1991.

[10] M. I. Miller, A. Trouvé, and L. Younes, "Geodesic shooting for computational anatomy," *Journal of mathematical imaging and vision*, vol. 24, no. 2, pp. 209–228, 2006.
