# Supplementary material for: STalign: Alignment of spatial transcriptomics data using diffeomorphic metric mapping
Source: Nat Commun. 2023 Dec 8;14:8123. doi: 10.1038/s41467-023-43915-7 (PMC10709594; doi:10.1038/s41467-023-43915-7)
Supplement: Supplementary file 3 — Reporting Summary [file 41467_2023_43915_MOESM3_ESM.pdf]

nature portfolio

Corresponding author(s): Dr. Jean Fan and Dr. Daniel Tward

Last updated by author(s): Oct 26, 2023

Reporting Summary

Nature Portfolio wishes to improve the reproducibility of the work that we publish. This form provides structure for consistency and transparency in reporting. For further information on Nature Portfolio policies, see our Editorial Policies and the Editorial Policy Checklist.

Statistics

For all statistical analyses, confirm that the following items are present in the figure legend, table legend, main text, or Methods section.

n/a	Confirmed	
☐	☒	The exact sample size (*n*) for each experimental group/condition, given as a discrete number and unit of measurement
☐	☒	A statement on whether measurements were taken from distinct samples or whether the same sample was measured repeatedly
☐	☒	The statistical test(s) used AND whether they are one- or two-sided<br>*Only common tests should be described solely by name; describe more complex techniques in the Methods section.*
☒	☐	A description of all covariates tested
☐	☒	A description of any assumptions or corrections, such as tests of normality and adjustment for multiple comparisons
☒	☐	A full description of the statistical parameters including central tendency (e.g. means) or other basic estimates (e.g. regression coefficient) AND variation (e.g. standard deviation) or associated estimates of uncertainty (e.g. confidence intervals)
☐	☒	For null hypothesis testing, the test statistic (e.g. *F*, *t*, *r*) with confidence intervals, effect sizes, degrees of freedom and *P* value noted<br>*Give P values as exact values whenever suitable.*
☒	☐	For Bayesian analysis, information on the choice of priors and Markov chain Monte Carlo settings
☒	☐	For hierarchical and complex designs, identification of the appropriate level for tests and full reporting of outcomes
☐	☒	Estimates of effect sizes (e.g. Cohen's *d*, Pearson's *r*), indicating how they were calculated

Our web collection on statistics for biologists contains articles on many of the points above.

Software and code

Policy information about availability of computer code

Data collection	No data was collected. Only publicly available data was analyzed.
Data analysis	The STalign software package version 0.1.0 was used to analyze the data. The source code is publicly available at https://github.com/JEFworks-Lab/STalign. For expression based performance analysis of STalign alignment we used MERINGUE (v1.0) to calculate Moran's I to identify significantly spatially patterned genes. To identify cell-types in the Visium data, we applied STdeconvolve (v1.6.0). Transcriptional clustering analysis and cell type annotation was performed using the SCANPY package [version 1.9.1]. Statistical analyses were either performed by the R package stats version 4.2.1. or by the SciPy Python package, v1.11.

For manuscripts utilizing custom algorithms or software that are central to the research but not yet described in published literature, software must be made available to editors and reviewers. We strongly encourage code deposition in a community repository (e.g. GitHub). See the Nature Portfolio guidelines for submitting code & software for further information.

Data

Policy information about availability of data

All manuscripts must include a data availability statement. This statement should provide the following information, where applicable:

- Accession codes, unique identifiers, or web links for publicly available datasets
- A description of any restrictions on data availability
- For clinical datasets or third party data, please ensure that the statement adheres to our policy

All data that was aligned with STalign is publicly available.

MERFISH datasets are available on the Vizgen website for MERFISH Mouse Brain Receptor Map data release (https://info.vizgen.com/mouse-brain-map).

The Visium dataset is available on the 10X Datasets website for Spatial Gene Expression Dataset by Space Ranger 1.3.0 (https://www.10xgenomics.com/resources/datasets/adult-mouse-brain-ffpe-1-standard-1-3-0).

STARMAP Plus data is available on the Broad Single Cell Portal (https://singlecell.broadinstitute.org/single_cell/study/SCP1830/spatial-atlas-of-molecular-[...]pes-and-aav-accessibility-across-the-whole-mouse-brain).
Developing heart data is available on the Human Developmental Cell Atlas https://hdca-sweden.scilifelab.se/a-study-on-human-heart-development/ via ST_heart_all_detected_nuclei.RData from https://github.com/MickanAsp/Developmental_heart.

The Xenium dataset (In Situ Replicate 1) of a fresh frozen mouse brain coronal section is available on the 10X Datasets website for Mouse Brain Dataset Explorer (https://www.10xgenomics.com/products/xenium-in-situ/mouse-brain-dataset-explorer).

The two Xenium datasets (In Situ Replicate 1 and In Situ Replicate 2) of a single breast cancer FFPE tissue block are available on the 10X Datasets website for High resolution mapping of the breast cancer tumor microenvironment using integrated single cell, spatial and in situ analysis of FFPE tissue (https://www.10xgenomics.com/products/xenium-in-situ/preview-dataset-human-breast).

The CCF and brain region annotations are available from the Allen Brain Atlas API https://help.brain-map.org/display/mouseconnectivity/API .

The data generated in this study for performance analysis of STalign have been deposited in a Zenodo repository (https://doi.org/10.5281/zenodo.8384019)34.

Research involving human participants, their data, or biological material

Policy information about studies with human participants or human data. See also policy information about sex, gender (identity/presentation), and sexual orientation and race, ethnicity and racism.

Reporting on sex and gender	We did not perform sex- and gender-based analysis.
Reporting on race, ethnicity, or other socially relevant groupings	We did not use any socially constructed or socially relevant categorization variable.
Population characteristics	There are no covariate-relevant population characteristics of the human data to report because we did not perform covariate analysis on the human data.
Recruitment	The collection of the human sample is described in "High resolution mapping of the breast cancer tumor microenvironment using integrated single cell, spatial and in situ analysis of FFPE tissue" (https://doi.org/10.1101/2022.10.06.510405). No description of how participants were recruited is provided.
Ethics oversight	As described in "High resolution mapping of the breast cancer tumor microenvironment using integrated single cell, spatial and in situ analysis of FFPE tissue" (https://doi.org/10.1101/2022.10.06.510405), the human sample was obtained from Discovery Life Sciences.

Note that full information on the approval of the study protocol must also be provided in the manuscript.

Field-specific reporting

Please select the one below that is the best fit for your research. If you are not sure, read the appropriate sections before making your selection.

[x] Life sciences [ ] Behavioural & social sciences [ ] Ecological, evolutionary & environmental sciences

For a reference copy of the document with all sections, see nature.com/documents/nr-reporting-summary-flat.pdf

Life sciences study design

All studies must disclose on these points even when the disclosure is negative.

Sample size	No statistical methods were used to predetermine the number of datasets involved in the study. We perform alignments using 17 datasets to demonstrate applications of our software tool STalign. These datasets are sufficient for the analyses as we are providing examples of application and not making claim of statistical significance of the utility of STalign for these types of datasets.<br><br>For our statistical tests on gene expression, cell type, and brain region correspondence, no sample-size calculations were performed. For comparing cosine similarity across non-spatially patterned genes (n=192) and spatially patterned genes (n=457), the sample sizes chosen were all genes of that type.<br><br>For statistical analysis of cell type composition of STalign-annotated brain regions and randomly demarcated brain regions of matched size, sample size was n=141 brain regions, all brain regions that are present across tissue replicates.

	For statistical analysis of entropy of cell type composition for expanded STalign-annotated brain regions and randomly demarcated brain regions of matched size, sample size was n=148 brain regions, all brain regions annotated in the tissue analyzed.
Data exclusions	Given that the MERFISH tissue section is larger than the Visium, we considered the aligned region to be limited to the MERFISH tissue that had a matching probability > 0.85 and restricted the list of cells in the aligned MERFISH dataset to only those in this region.<br><br>To aggregate the cells in the aligned MERFISH dataset into pseudospots that match with the Visium spots, we restricted to classifying cells as within the pseudospot that corresponds to the Visium spot if the distance of the cell to the Visium centroid was less than the Visium spot radius.<br><br>For gene expression correspondence analysis, we restricted to the 415 genes that had at least one copy in both the MERFISH and Visium datasets and that were detected in more than one spot in the Visium dataset.<br><br>To identify cell-types in the Visium data, we applied STdeconvolve on a corpus of 838 genes after filtering out lowly expressed genes (<100 copies), genes present in < 5% of spots and genes present in > 95% of spots and restricting to significantly over-dispersed with alpha =1e-16 to obtain a corpus < 1000 genes, resulting in 16 deconvolved cell-types.<br><br>To match deconvolved cell-types from Visium and single-cell clusters from MERFISH, we restricted to the 257 shared genes and correlated the resulting normalized transcriptional profiles using Spearman correlation. For evaluating alignment of matched cell-type, we considered a Visium deconvolved cell-type and MERFISH single-cell cluster as a match if they had transcriptional similarity > 0.5.
Replication	While we demonstrate application of our software tool through 23 distinct alignments on different tissues from different technologies, we did not perform any biological experiments so we have no experimental replicates.
Randomization	For the statistical analyses perform in this study, samples were not allocated in groups at random. For comparing cosine similarity across non-spatially patterned genes and spatially patterned genes, genes were allocated into these groups by calculate Moran's I and genes with an adjusted p-value < 0.05 were identified as significantly spatially patterned genes. For comparing cell type composition of STalign-annotated brain regions and randomly demarcated brain regions of matched size, the brain regions in the former group are all true brain regions annotated and the brain regions in the latter group are faux brain regions that were generated to be used like a null. Randomized allocation into groups is not relevant to this study as random allocations would not fit the definitions of the groups compared and there are no covariates for which to control.
Blinding	Blinding is not relevant to any of the statistical analyses perform in this study. Since we simply report the p-values of Wilcoxon rank-sum test, paired t-test, and Mann-Whitney tests indicating whether the difference between groups are statistically significant of not, blinding to allocation between groups for would not affect interpretation of the results.

Reporting for specific materials, systems and methods

We require information from authors about some types of materials, experimental systems and methods used in many studies. Here, indicate whether each material, system or method listed is relevant to your study. If you are not sure if a list item applies to your research, read the appropriate section before selecting a response.

Materials & experimental systems

n/a	Involved in the study	
☒	☐	Antibodies
☒	☐	Eukaryotic cell lines
☒	☐	Palaeontology and archaeology
☒	☐	Animals and other organisms
☒	☐	Clinical data
☒	☐	Dual use research of concern
☒	☐	Plants

Methods

n/a	Involved in the study	
☒	☐	ChIP-seq
☒	☐	Flow cytometry
☒	☐	MRI-based neuroimaging
